# Successful management of concurrent COVID-19 and *Pneumocystis Jirovecii* Pneumonia in kidney transplant recipients: a case series

**DOI:** 10.1186/s12890-023-02764-2

**Published:** 2023-11-21

**Authors:** Guoping Li, Daxi Ji, Youcheng Chang, Zheng Tang, Dongrui Cheng

**Affiliations:** 1 Department of Nephrology, Nanjing Yimin Hospital, Nanjing, 211100 China; 2 National Clinical Research Center of Kidney Diseases, Jinling Hospital, Nanjing University School of Medicine, Nanjing, 200016 China

**Keywords:** Kidney transplant recipients, COVID-19, Pneumocystis Pneumonia, *Pneumocystis Jirovecii*, Co-infection

## Abstract

**Background:**

Pneumocystis pneumonia (PCP) is a life-threatening pulmonary fungal infection that predominantly affects immunocompromised individuals, including kidney transplant recipients. Recent years have witnessed a rising incidence of PCP in this vulnerable population, leading to graft loss and increased mortality. Immunosuppression, which is essential in transplant recipients, heightens susceptibility to viral and opportunistic infections, magnifying the clinical challenge. Concurrently, the global impact of coronavirus disease 2019 (COVID-19), caused by severe acute respiratory syndrome coronavirus 2 (SARS-CoV-2), has been profound. Kidney transplant recipients have faced severe outcomes when infected with SARS-CoV-2, often requiring intensive care. Co-infection with COVID-19 and PCP in this context represents a complex clinical scenario that requires precise management strategies, involving a delicate balance between immunosuppression and immune activation. Although there have been case reports on management of COVID-19 and PCP in kidney transplant recipients, guidance on how to tackle these infections when they occur concurrently remains limited.

**Case presentations:**

We have encountered four kidney transplant recipients with concurrent COVID-19 and PCP infection. These patients received comprehensive treatment that included adjustment of their maintenance immunosuppressive regimen, anti-pneumocystis therapy, treatment for COVID-19 and other infections, and symptomatic and supportive care. After this multifaceted treatment strategy, all of these patients improved significantly and had favorable outcomes.

**Conclusions:**

We have successfully managed four kidney transplant recipients co-infected with COVID-19 and PCP. While PCP is a known complication of immunosuppressive therapy, its incidence in patients with COVID-19 highlights the complexity of dual infections. Our findings suggest that tailored immunosuppressive regimens, coupled with antiviral and antimicrobial therapies, can lead to clinical improvement in such cases. Further research is needed to refine risk assessment and therapeutic strategies, which will ultimately enhance the care of this vulnerable population.

## Background

Pneumocystis pneumonia (PCP) is a severe opportunistic pulmonary fungal infection caused by *Pneumocystis jirovecii*, which usually occurs in immunocompromised patients, especially those infected with human immunodeficiency virus. In recent years, PCP has become increasingly prevalent among solid organ transplant recipients, particularly kidney transplant recipients, and individuals with hematological malignancies [1–3]. Notably, PCP has been associated with a heightened risk of graft loss and mortality [4]. Kidney transplant recipients require maintenance immunosuppressive therapy and therefore have increased susceptibility to both viral and opportunistic infections.

Coronavirus disease 2019 (COVID-19), caused by severe acute respiratory syndrome coronavirus 2 (SARS-CoV-2), has posed an unprecedented threat to global health. By August 2023, a total of 769,806,130 cases of COVID-19, including 6,955,497 deaths, had been confirmed worldwide [5]. COVID-19 infection in kidney transplant recipients may be particularly severe and require admission for intensive care [6].

In clinical practice, kidney transplant recipients with COVID-19 and PCP co-infection represent a multifaceted and intricate clinical scenario. Effective management of this dual infection necessitates precise clinical strategies, frequently involving a delicate balance between immunosuppression and immune activation [7]. While a number of case reports have documented the management of either COVID-19 infection or PCP infection in kidney transplant recipients [6, 8], the literature on the management of these infections when they occur simultaneously is limited.

This report describes the successful clinical management of COVID-19 and PCP co-infection in four kidney transplant recipients. Our intention is to provide health care practitioners with valuable insights and potential guidance for effective management of similar cases, with the ultimate goal of improving patient outcomes.

## Case presentations

### Case 1

**Fig. 1:**
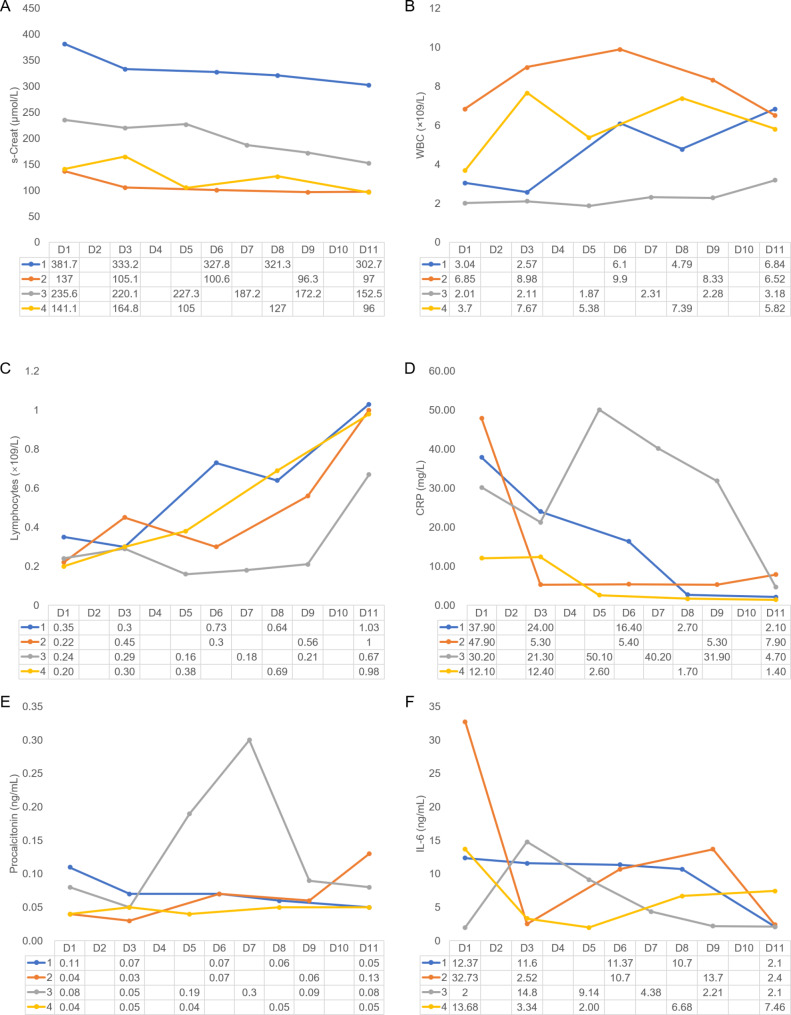
Clinical laboratory results. AWBC, white blood cells; CRP, C-reactive Protein; IL-6, interleukin-6; Tac, tacrolimus; MMF, mycophenolate mofetil

A 65-year-old man with IgA nephropathy and chronic renal insufficiency secondary to end-stage renal failure (ESRF) underwent living donor kidney transplantation in 2011. His renal function recovered well after the operation. Serum creatinine was maintained at 200–300 µmol/L, and immunosuppression was maintained using a triple-drug regimen consisting of mycophenolate mofetil (MMF) 0.5 g twice daily, tacrolimus 1 mg twice daily, and prednisone 5 mg once daily. On January 25, 2023, the patient developed fever, cough, chest tightness, and fatigue following physical activity, leading to hospitalization on February 2, 2023. On admission, he had a body temperature of 39.2 °C and a blood oxygen saturation of 90% in ambient air. A quantitative reverse transcription-PCR (RT-qPCR) assay for SARS-CoV-2 was positive (Table 1). The pertinent admission-related test results are shown in Fig. 1.

**Table 1:** Patient Characteristics and Treatment Options

Variables	Case 1	Case 2	Case 3	Case 4
Sex	M	M	M	F
Age (years)	65	27	52	66
Months post-Tx	144	27	79	81
**Vital signs**				
SpO_2_	90%	91%	89%	89%
**Signs/symptoms**				
Fever (T > 37.5 °C)	39.2℃	39.0℃	39.7℃	39.0℃
Cough	Yes	Yes	Yes	Yes
Diarrhea	Yes	Yes	Yes	Yes
Chest tightness	Yes	Yes	Yes	Yes
Weak	Yes	Yes	Yes	Yes
CT (Lung infection)	Yes	Yes	Yes	Yes
Immunosuppressive Regimen at admission	MMF/FK/PDN	MMF/FK/PDN	MMF/FK/PDN	MMF/FK/PDN
Treatment	MPSL/ NTV/RIT/ GCV/ SMX/ CAS/ MXF	MPSL/ NTV/RIT/ GCV/ SMX/ CAS/ MXF	MPSL/ NTV/RIT/ GCV/ SMX/ CAS/ MXF	MPSL/ NTV/RIT/ GCV/ SMX/ CAS/ MXF

CT, Computerized Tomography; MMF, mycophenolate mofetil; FK, tacrolimus; PDN, prednisone; MPSL, Methylprednisolone; NTV/RIT, Naimatevir/Ritonavir combination; GCV, Ganciclovir; SMX, Sulfamethoxazole; CAS, Caspofungin; MXF, Moxifloxacin

**Fig. 2:**
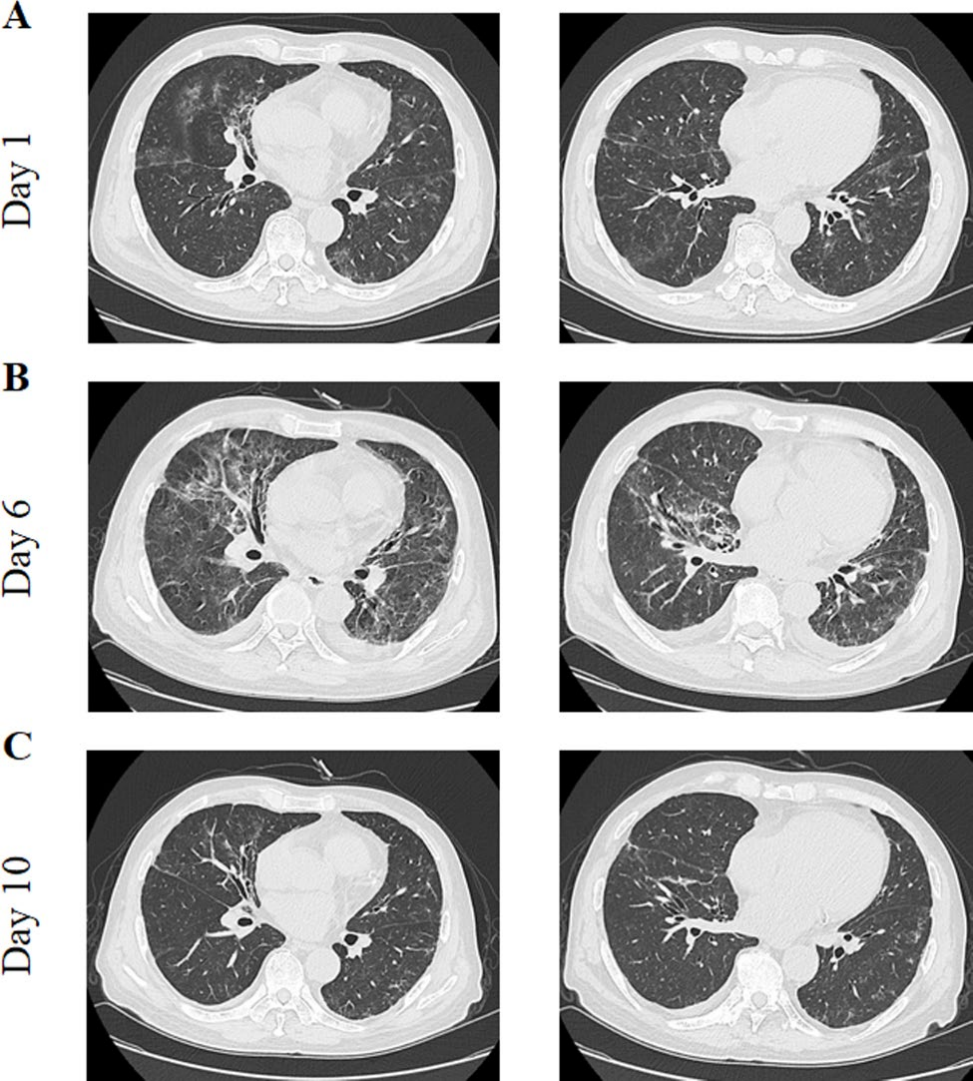
Case 1-Computed tomography images at different hospital days

A computed tomography (CT) scan of the chest revealed multiple patchy small nodular ground-glass opacities in both lungs, which were accompanied by grid-like and scattered fibrous cord-like opacities with indistinct borders (Fig. 2). Subsequently, a 5-mL sample of bronchoalveolar lavage fluid was collected and send to the local microbiology laboratory for metagenomic next-generation sequencing, which confirmed *P. jirovecii* infection (Table 2). As part of the treatment strategy, the patient’s maintenance immunosuppressive regimen was discontinued. The therapeutic protocol consisted of methylprednisolone 40 mg as the sole anti-rejection agent, with antiviral intervention that consisted of namatevir/ritonavir (namatevir 300 mg/ritonavir 100 mg twice daily on day 1; namatevir 150 mg/ritonavir 100 mg once daily on days 2–5) and ganciclovir 250 mg/day. The patient was also started on an antimicrobial regimen of sulfamethoxazole (administered as 3 tablets per dose, three times daily), caspofungin (70 mg on day 1, followed by 50 mg/day), and moxifloxacin 250 mL (Table 1).

**Table 2:** Microbiological Outcome Criteria

Pathogens	Case 1	Case 2	Case 3	Case 4
SARS-CoV-2 RNA	**Positive**	**Positive**	**Positive**	**Positive**
mNGS Sample	BALF	Blood	Blood	-
Pneumocystis jirevocii DNA	**Positive**	**Positive**	**Positive**	-
Human betaherpesvirus 5 DNA	**Positive**	**Positive**	Negative	-
Human alphaherpesvirus 1 DNA	**Positive**	Negative	**Positive**	-
Human gammaherpesvirus 4 DNA	**Positive**	Negative	Negative	-
Human polyomavirus 1 DNA	Negative	**Positive**	Negative	-
Aspergillus DNA	**Positive**	Negative	Negative	-

mNGS, metagenomic next-generation sequencing; BALF, Bronchoalveolar lavage fluid

After 4 days of treatment, the patient tested negative for COVID-19 and his temperature returned to normal. Seven days later, his symptoms of cough and chest tightness had improved slightly, vital signs were stable, and his blood oxygen saturation was above 95% on supplemental oxygen at a flow rate of 3 L/min. Chest CT showed that the lesions were partially absorbed (Fig. 2). Methylprednisolone was stopped, prednisone acetate was administered orally, and tacrolimus was added. Finally, when the absolute value of lymphocytes is greater than 1000, added MMF. Thereafter, his vital signs were stable, his symptoms improved, and he was discharged from hospital. The relevant test results and changes during hospitalization are summarized in Fig. 1, and the changes on chest CT are shown in Fig. 2.

### Case 2

**Fig. 3:**
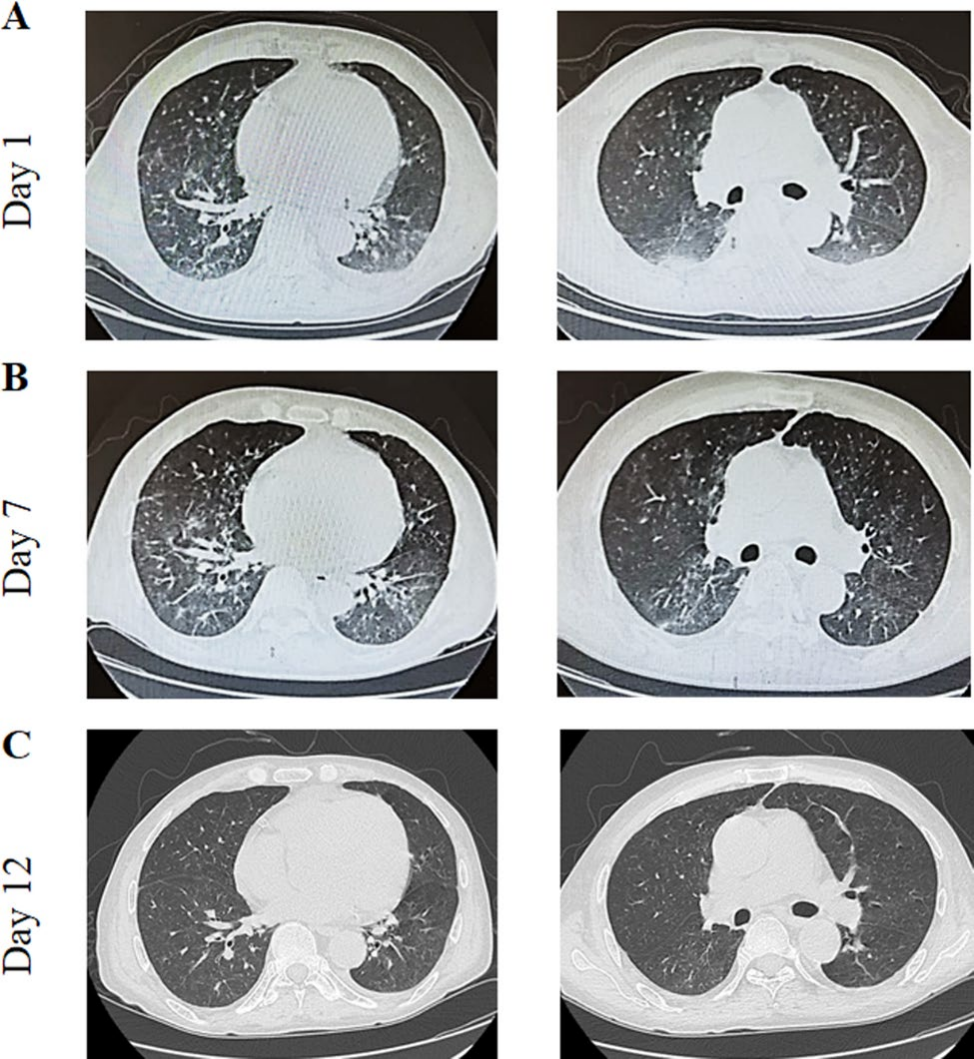
Case 2-Computed tomography images at different hospital days

A 27-year-old man underwent living donor kidney transplantation for hypertensive nephropathy-associated ESRF in 2020. Following the procedure, his renal function recovered well, with serum creatinine levels consistently maintained within the normal range. Immunosuppression was maintained by a triple-drug regimen consisting of MMF 0.36 g twice daily, tacrolimus 2 mg in the morning and 1 mg in the evening, and prednisone 5 mg once daily. On March 15, 2023, the patient presented with fever, cough, chest tightness, and shortness of breath, prompting hospitalization on March 28, 2023. On admission, he had a body temperature of 39.0 °C and a blood oxygen saturation of 91% in ambient air (Table 1). Admission-related test results are shown in Fig. 1. An RT-qPCR assay for SARS-CoV-2 was positive (Table 2). Chest CT scans revealed multiple patchy nodular ground-glass opacities in both lungs, accompanied by grid-like and scattered fibrous cord-like opacities with blurred borders (Fig. 3). Next-generation sequencing of pathogenic microorganisms in peripheral blood revealed *P. jirovecii* infection (Table 2).

The therapeutic protocol consisted of methylprednisolone 40 mg as the sole anti-rejection agent, with antiviral intervention that consisted of namatevir/ritonavir (namatevir 300 mg/ritonavir 100 mg twice daily on days 1–5) and ganciclovir 250 mg/day. The remaining treatment protocols are the same as those in case 1. (Table 1). Following a 4-day course of treatment, a COVID-19 test was negative and the patient’s body temperature had returned to the normal range. Seven days thereafter, notable relief of symptoms, including cough and chest tightness, was observed and vital signs were stable. The patient consistently achieved a blood oxygen saturation higher than 95% on supplemental oxygen at a flow rate of 3 L/min. Subsequent chest CT demonstrated partial resolution of the previously identified lesions. The patient’s vital signs remained stable, and symptomatic improvement subsequently continued under the same treatment protocol. The examination outcomes and changes observed during hospitalization are shown in Fig. 1 and findings on chest CT over time in Fig. 2.

### Case 3

**Fig. 4:**
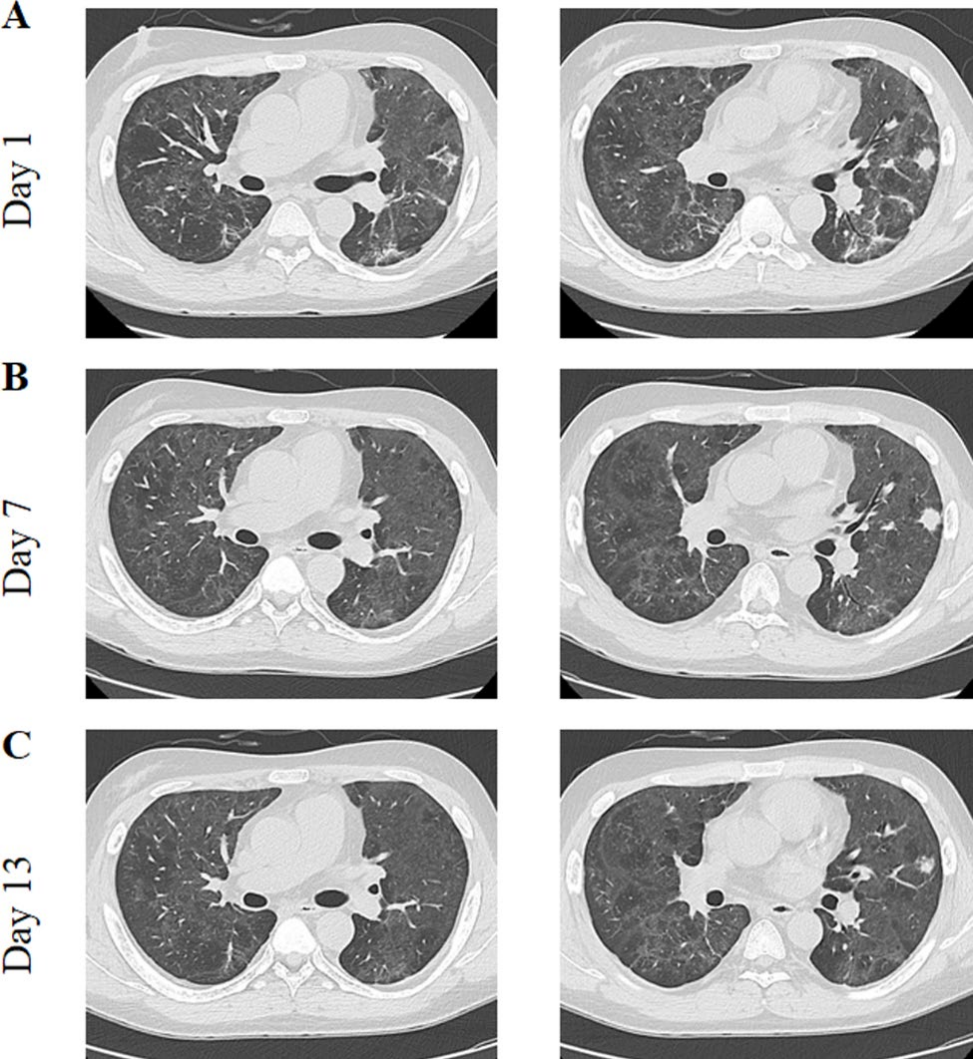
Case 3-Computed tomography images at different hospital days

A 52-year-old man with ESRF associated with polycystic kidney disease underwent living donor kidney transplantation in 2016. Following the procedure, his renal function showed marked recovery. His serum creatinine level was maintained within the range of 120–160 µmol/L. Immunosuppression was maintained using a triple-drug regimen consisting of MMF (0.75 g in the morning, 0.5 g in the evening), tacrolimus (2 mg in the morning, 1 mg in the evening), and prednisone (5 mg once daily). On April 15, 2023 he developed symptoms of fever, cough, chest tightness, and fatigue post-exertion, leading to hospitalization on April 19, 2023. On admission, his body temperature was 39.7 °C and his blood oxygen saturation was 89% in ambient air. His admission-related test outcomes are summarized in Fig. 1. An RT-qPCR assay for SARS-CoV-2 was positive, as shown in Table 2. Chest CT scans revealed multiple patchy grid-like and cord-like shadows characterized by increased density in both lungs with blurred boundaries (Fig. 4). Next-generation sequencing of pathogenic microorganisms in peripheral blood confirmed *P. jirovecii* infection (Table 2).

After 2 days using the same treatment plan as case 1 (Table 1), the patient’s COVID-19 test was negative and his body temperature had returned to the normal range. Five days later, his symptoms of cough and chest tightness had resolved, and vital signs were stable. His blood oxygen saturation was consistently above 96% on supplemental oxygen at a flow rate of 3 L/min. A chest CT scan showed partial absorption of the lesions. The follow-up treatment plan was then initiated, and the patient’s vital signs remained stable, his symptoms improved, and he was finally discharged. Figure 1 shows the relevant test results and changes observed during hospitalization, while the chest CT findings over time are shown in Fig. 4.

### Case 4

**Fig. 5:**
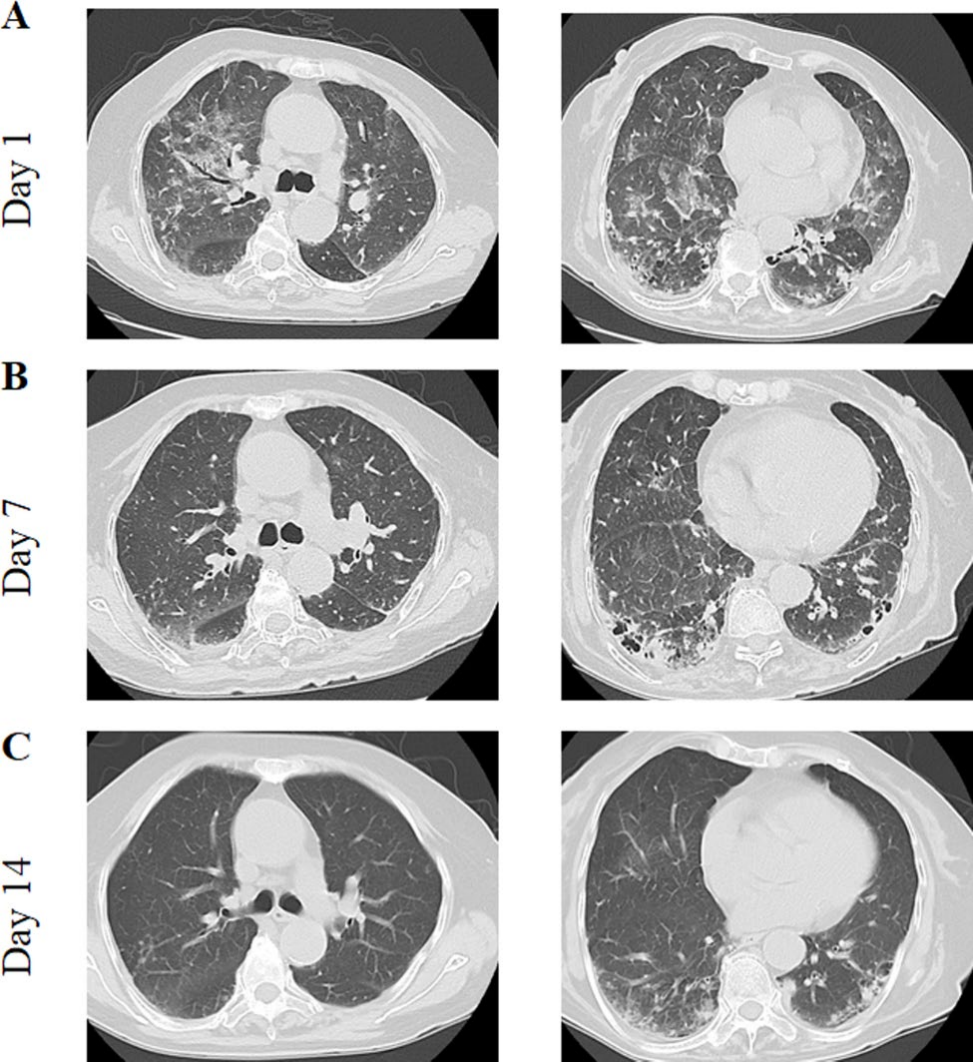
Case 4-Computed tomography images at different hospital days

A 66-year-old woman underwent living donor kidney transplantation for ESRF stemming from polycystic kidney disease in 2016. Her renal function recovered well after surgery, with serum creatinine levels that were consistently within the normal range. Immunosuppression was maintained using a triple-drug regimen consisting of MMF 0.75 g twice daily, tacrolimus 1 mg twice daily, and prednisone 5 mg once daily. On May 25, 2023, the patient developed symptoms of fever, cough, chest tightness, and fatigue, prompting hospitalization on June 1, 2023. The relevant admission-related test outcomes are summarized in Fig. 1. An RT-qPCR assay for SARS-CoV-2 was positive. Chest CT scans revealed multiple patchy ground-glass density shadows alongside grid-like shadows characterized by blurred edges involving both lungs (Fig. 5). In view of financial constraints, next-generation sequencing was not performed for this patient.

Drawing on the experience at our institution, a 3-day adherence to the identical treatment plan as case 2 (Table 1) resulted in conversion of her COVID-19 test result from positive to negative with normalization of body temperature, thus confirming the efficacy of treatment. A week later, her symptoms of cough and chest tightness were alleviated and her vital signs were stable. Her blood oxygen saturation was consistently above 97% on supplemental oxygen at a flow rate of 3 L/min. Subsequent CT scans of the chest indicated partial absorption of the identified lesions. Continued adherence to the follow-up treatment protocol led to sustained improvement, culminating in the patient’s discharge from hospital. The results of relevant tests and changes noted during hospitalization are shown in Fig. 1 and progression of the chest CT findings in Fig. 5.

## Discussion and conclusions

Thus far, there have been three reported cases of kidney transplant recipients who developed COVID-19 with concurrent PCP infection. Two of these cases were successfully treated and one was ultimately fatal [1–3]. In this report, we describe four kidney transplant recipients (three male, one female) in Nanjing, China who were admitted with COVID-19 and PCP co-infection that was managed successfully. The mean patient age was 53 years, and the average interval between kidney transplantation and onset of PCP was 83 months. PCP is a significant complication arising from immunosuppressive therapy in individuals who have undergone solid organ transplantation. Trimethoprim/sulfamethoxazole has been used widely for prophylaxis against PCP; however, its potential risks and adverse effects outweigh its preventive benefit [9]. Therefore, trimethoprim/sulfamethoxazole is generally not recommended for prophylaxis against PCP nowadays. Moreover, a recent report suggests that patients on MMF may not need PCP prophylaxis [10]. All our four cases received a combined immunosuppressive regimen of MMF, tacrolimus, and prednisone following kidney transplantation. However, it is noteworthy that these individuals developed PCP infection at varying time intervals after transplantation. It may be attributed to the SARS-CoV-2 infection, as evidenced by an unexpectedly high proportion of PCP samples in critically ill patients with COVID-19 [11].

All four cases were confirmed to have SARS-CoV-2 infection by RT-qPCR on admission. Furthermore, three of these four patients were diagnosed with PCP by metagenomic next-generation sequencing, with two found to have co-infection with cytomegalovirus. All four cases were found to have lymphocytopenia on admission, with absolute lymphocyte counts of less than 500 × 10^6^ cells/L; the risk of developing PCP was 18.7-fold greater in these patients than in those with an absolute lymphocyte count higher than 500 × 10^6^ cells/L [12]. The patients’ maintenance immunosuppressive regimens were discontinued to enhance the immune response to the infections. In all cases, antiviral (namatevir/ritonavir/ganciclovir) and antimicrobial (caspofungin/sulfamethoxazole/moxifloxacin) therapy was administered to address PCP and other infections. Methylprednisolone had been administered to control the inflammatory response and alleviate respiratory symptoms. After an average of 6 days of combination therapy, all four patients showed improvements in their clinical symptoms, with conversion to a negative COVID-19 test result. The limitation of this case series is that chest CT was used to diagnose PCP in one of the cases. Bilateral diffuse ground-glass opacities with interstitial infiltrates are typical findings in PCP [13–16]. After treatment, the ground-glass opacities partially resolved in all cases. Upon observing a positive response to treatment, the decision was made to reinstate the maintenance immunosuppressive regimen in order to minimize the risk of rejection.

In conclusion, this case series provides a foundation for further research in the field of simultaneous COVID-19 and PCP infection in kidney transplant recipients. Continued investigation of the risk factors, optimal treatment approaches, and long-term outcomes is essential for improvement of the management and care of these complex cases.

## Data Availability

All data generated or analyzed during this study are included in this published article.
